# Ag85A-PEGylated Propolis Nanoparticles Exhibit Intracellular Antimycobacterial and Host-Protective Activities Against *Mycobacterium tuberculosis*

**DOI:** 10.3390/ijms27167223

**Published:** 2026-08-13

**Authors:** Sanonthinee Sookkree, Sirikwan Sangboonruang, Ponrut Phunpae, Siriwan Thaisakun, Narumon Phaonakrop, Sittiruk Roytrakul, Khajornsak Tragoolpua

**Affiliations:** 1 Division of Clinical Microbiology, Department of Medical Technology, Faculty of Associated Medical Sciences, Chiang Mai University, Chiang Mai 50200, Thailand; sanonthinee_sookkree@cmu.ac.th (S.S.); sirikwan.sang@cmu.ac.th (S.S.); ponrut.p@cmu.ac.th (P.P.); 2 Infectious Diseases Research Unit (IDRU), Faculty of Associated Medical Sciences, Chiang Mai University, Chiang Mai 50200, Thailand; 3 National Center for Genetic Engineering and Biotechnology, National Science and Technology for Development Agency, Pathum Thani 12120, Thailand; siriwan.tha@ncr.nstda.or.th (S.T.); narumon.pha@biotec.or.th (N.P.); sittiruk@biotec.or.th (S.R.)

**Keywords:** *Mycobacterium tuberculosis*, ethanolic extract of propolis, nanodelivery system, antimycobacterial activity, host protective immune response, proteomics analysis

## Abstract

Tuberculosis (TB), caused by the intracellular pathogen *Mycobacterium tuberculosis* (Mtb), remains a major global health challenge. The prolonged duration of treatment and the emergence of multidrug-resistant strains have highlighted the need for alternative therapeutic strategies. This study investigated the therapeutic potential of Ag85A aptamer-conjugated PEGylated niosomes encapsulating ethanolic extract of propolis (Ag85A-PEGNio/EEP) using Mtb-infected macrophage model. Ag85A-PEGNio/EEP exhibited efficient cellular uptake, with more than 99.8% internalization by macrophages, and trafficked host phagolysosome, facilitating targeted delivery of EEP to intracellular Mtb. Ag85A-PEGNio/EEP treatment showed an anti-mycobacterium efficacy by reducing intracellular Mtb viability by approximately 51.2% compared with untreated controls. Moreover, Ag85A-PEGNio/EEP modulated macrophage immune responses by significantly increasing the expression of the pro-inflammatory cytokines *IL-12* (8.4-fold) and *IL-6* (3.8-fold), while markedly decreasing the expression of the anti-inflammatory cytokine *IL-10* (6.4-fold). Protein–protein interaction (PPI) network analysis further revealed the association of proteins with immune regulation and antioxidant responses in treated cells. These findings suggest that Ag85A-PEGNio/EEP functions as a dual-action therapeutic platform by enhancing intracellular anti-mycobacterial activity while balancing host immune responses. This targeted nano-delivery system represents a promising candidate for host-directed TB therapy and further investigations are needed to validate these outcomes and explore their potential applications against TB treatment challenges.

## 1. Introduction

*Mycobacterium tuberculosis* (Mtb), the causative intracellular pathogen of tuberculosis (TB), continues to pose a global health burden, with approximately 10 million new cases and 1.3 million deaths reported annually [1]. Transmission occurs through the inhalation of aerosolized droplets containing viable bacilli. Following deposition in the alveolar spaces, Mtb is recognized by the different types of pattern recognition receptors (PRRs), including Toll-like receptors (TLRs), on resident alveolar macrophages and is subsequently internalized by phagocytosis. Beyond their role in pathogen clearance, macrophages serve as antigen-presenting cells that process and present mycobacterial antigens to T-lymphocytes, while coordinating the recruitment and activation of additional immune cells at the site of infection. This immune response promotes either bacterial eradication or the development of granuloma formation [2]. Notably, macrophages represent a critical cellular niche for Mtb, where the outcome of infection is determined by the dynamic interplay between host antimicrobial mechanisms and bacterial survival strategies.

Upon encountering intracellular Mtb, macrophages initiate diverse bactericidal mechanisms, including phagosome–lysosome fusion, apoptosis, and the production of cytokines [3]. Alveolar macrophages attempt to eliminate the pathogen via the secretion of proteolytic enzymes and pro-inflammatory cytokines, such as tumor necrosis factor-alpha (TNF-α), interleukin 1-beta (IL-1β), interleukin 6 (IL-6), and interferon-gamma (IFN-γ). Additionally, they generate reactive oxygen species (ROS) and the activate inducible nitric oxide synthase (iNOS) to synthesize nitric oxide (NO) [3,4]. However, Mtb employs distinct virulence factors to evade these host defenses, facilitating intracellular replication by modifying TLRs signalings, arresting phagosome maturation, and modulating host cell death [5].

The World Health Organization (WHO) recommends a standard regimen for drug-susceptible TB comprising four first-line antibiotics: isoniazid (INH), rifampicin (RIF), pyrazinamide (PZA) and ethambutol (EMB) [1]. However, poor patient adherence due to adverse effects, prolonged treatment timelines, and antibiotic misuse have collectively driven the emergence of drug-resistant TB strains [6]. To combat resistance, the development of bedaquiline, delamanid and pretomanid over the past decade represents a significant therapeutic advance. These agents exhibit potent antibacterial activity against both actively replicating and dormant bacilli due to their superior macrophage penetration [7]. However, their deployment is strictly reserved for rifampicin-resistant (RR-TB) or multidrug-resistant tuberculosis (MDR-TB). Furthermore, their clinical utility is limited due to high costs in low-to-middle-income countries (LMICs) and risks of cardiotoxicity and hematological toxicities [8]. Given that successful eradication depends on the ability of TB drugs to infiltrate complex lung lesions and penetrate host macrophage membranes [9]. Thus, alternative strategies to target intracellular Mtb are urgently required.

Natural products are increasingly considered an attractive therapeutic alternative for various diseases due to their low toxicity, cost-effectiveness, and high efficacy. Propolis, a natural resinous wax-like substance produced by honeybees (*Apis mellifera*), contains bioactive compounds including alkaloids, flavonoids, and terpenoids that have demonstrated anti-mycobacterial activity [10,11]. Notably, propolis extracts have been shown to inhibit Mtb growth directly and exhibit synergistic effects with first-line anti-TB drugs such as isoniazid, rifampicin, and streptomycin [12]. To enhance its therapeutic delivery, nano-platforms have recently been explored. Previously, an Ag85A aptamer conjugated PEGylated niosomal formulation encapsulating ethanolic extract of propolis (Ag85A-PEGNio/EEP) was developed, demonstrating targeted binding to Mtb and potent anti-mycobacterial activity [13]. Nonetheless, further investigation is required to evaluate the efficacy of Ag85A-PEGNio/EEP against intracellular Mtb within host infection models, as well as its subsequent impact on host immune responses.

Here, we established an in vitro macrophage infection model to evaluate the potential of Ag85A-PEGNio/EEP as a novel therapeutic candidate. We hypothesized that this nano-formulation would exhibit potent anti-intracellular mycobacterial activity. Furthermore, we investigated the resulting cellular responses and immunomodulatory pathways triggered by Ag85A-PEGNio/EEP using a proteomics-based approach. In conclusion, these findings highlight the potential of Ag85A-PEGNio/EEP as a promising candidate for further development in alternative pulmonary tuberculosis therapies.

## 2. Results

### 2.1. Intracellular Uptake and Localization of Ag85A-PEGNio/EEP in Macrophage

**Figure 1:**
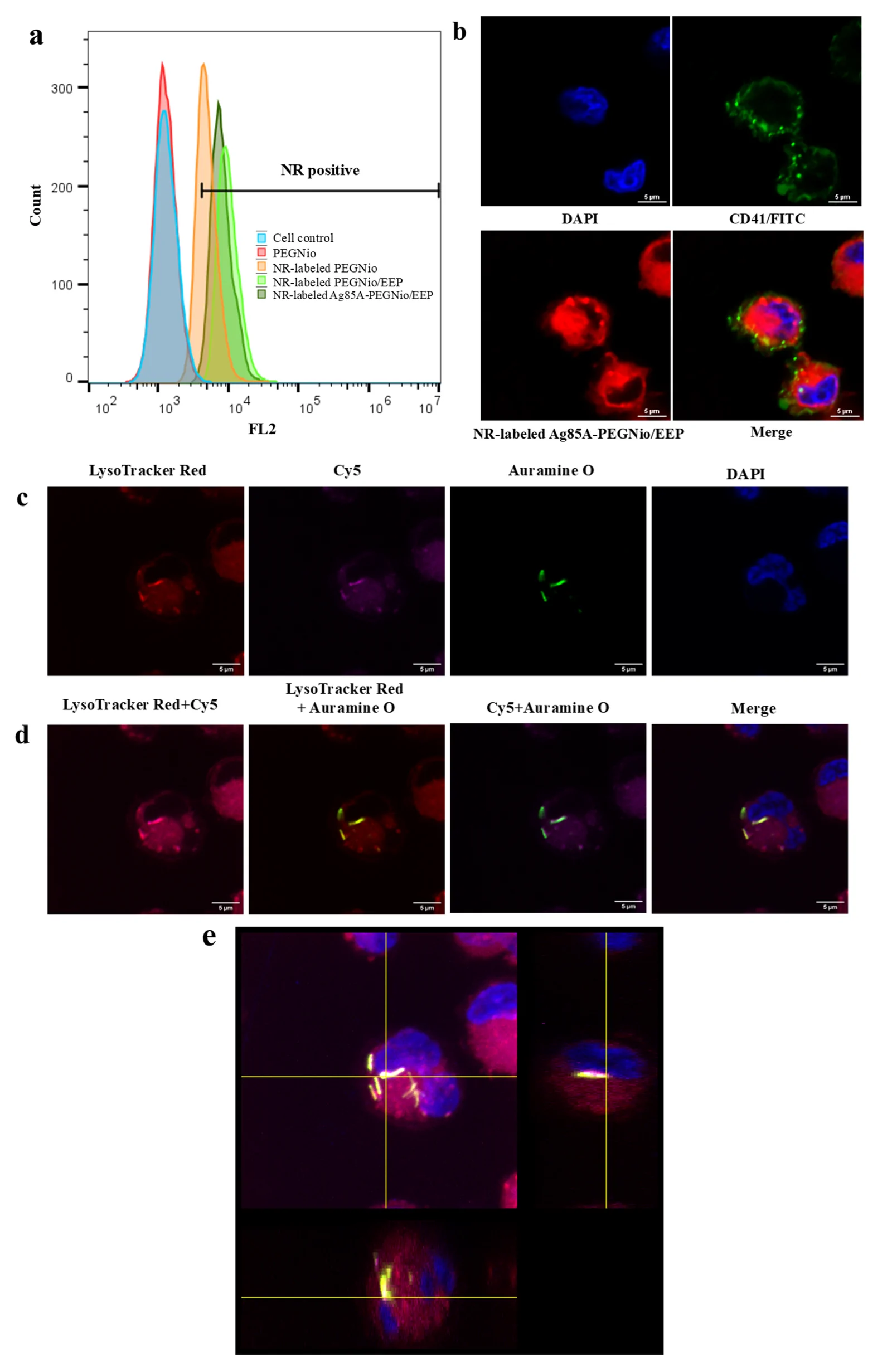
Cellular uptake and localization of Ag85A-PEGNio/EEP in Mtb-infected macrophages. (**a**) Flow cytometric detection of PEGNio, PEGNio/EEP, and Ag85A-PEGNio/EEP uptake by NR8383 cells after incubation for 2 h. (**b**) NR-labeled Ag85A-PEGNio/EEP (red) was localized in the cytoplasm of the cells, indicated by CD41/FITC-stained cell surface (green) and nuclei-stained DAPI (blue). (**c**) Individual fluorescence channels demonstrating host cell nuclei (DAPI, blue), phagolysosomes (LysoTracker, Red), Cy5-conjugated Ag85A-PEGNio/EEP (Cy5, magenta), and intracellular Mtb (Auramine O, green). (**d**) Dual-channel overlays and (**e**) orthogonal projection of confocal sections (Z-stack). Images were acquired using CLSM (FluoView FV3000; Olympus Optical, Tokyo, Japan). The scale bar represents 5 µm.

To confirm the internalization efficiency of Ag85A-PEGNio/EEP, intracellular uptake of Nile red-labeled Ag85A-PEGNio/EEP by NR8383 macrophages was quantified by flow cytometry. As shown in Figure 1a, 99.0% and 99.8% of macrophages internalized NR-labeled PEGNio/EEP and Ag85A-PEGNio/EEP, respectively, compared with unlabeled niosomes. Intracellular localization was further confirmed by CLSM. Macrophage membranes were stained with anti-CD41/FITC (Green), nuclei were counterstained with DAPI (blue), and NR-labeled niosomes were visualized in red. The result revealed that NR-labeled niosomes were uptaken by macrophages and predominantly distributed within the cytoplasm of macrophages (Figure 1b).

To further elucidate the intracellular trafficking of Ag85A-PEGNio/EEP, the localization of niosomes within Mtb-infected macrophages was investigated using CLSM. As shown in Figure 1c, Cy5-labeled niosomes (magenta) were successfully internalized by the NR8383 macrophages and distributed throughout the cytoplasmic region of cells. To determine their intracellular fate tracking, phagolysosomes were stained with LysoTracker Red. The colocalization of Cy5 fluorescence with LysoTracker Red produced a bright pink signal, indicating that the internalized niosomes were trafficked to phagolysosomal compartments following phagocytic uptake (Figure 1d). Simultaneously, Auramine O-stained Mtb bacilli (green) exhibited substantial colocalization with LysoTracker Red, generating a yellow fluorescence signal, which confirmed the presence of intracellular bacteria within phagolysosomal compartments of infected NR8383 macrophages.

Importantly, the overlap between the Cy5-labeled niosomes and Auramine O-stained Mtb signals was observed within the phagolysosomal compartment, demonstrating that both the niosomes and intracellular bacteria were localized to the same subcellular region. The colocalization of Cy5-labeled niosomes, LysoTracker Red-stained phagolysosomes, and Auramine O-labeled Mtb further indicated that Ag85A-PEGNio/EEP was successfully delivered to the intracellular site of Mtb infection (Figure 1e). This targeted localization supports the potential of Ag85A-PEGNio/EEP to enhance the delivery of therapeutic agents directly to intracellular mycobacterial reservoirs.

### 2.2. Anti-Intracellular Mycobacterial Activity of Ag85A-PEGNio/EEP

**Figure 2:**
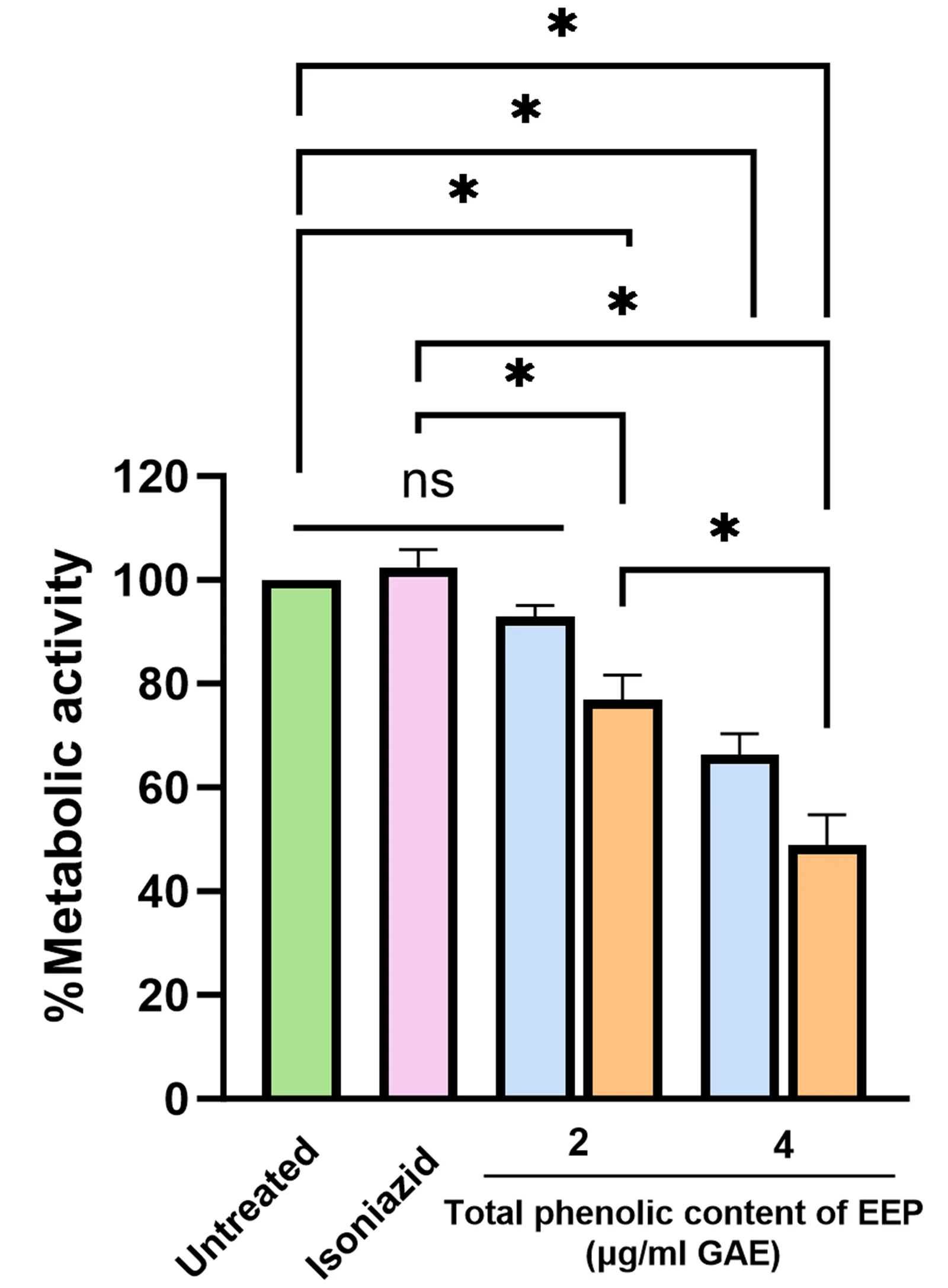
Anti-intracellular mycobacterial activity of Ag85A-PEGNio/EEP. All values are expressed as the mean ± SEM of three independent experiments performed in triplicate. * *p* < 0.05 when compared to each group. ns: not significant when compared to each group. Green, pink, blue and orange bars represent Untreated control, isoniazid treatment, Ag85A-PEGNio treatment and Ag85A-PEGNio/EEP treatment, respectively.

The viability of intracellular Mtb was evaluated using the REMA. As shown in Figure 2, treatment with INH at the critical concentration of 1 µg/mL showed no significant reduction in the metabolic activity of intracellular Mtb compared with the untreated control. Similarly, treatment with Ag85A-PEGNio control at 2 µg/mL gallic acid equivalent (GAE) showed no significant effect on bacterial viability. In contrast, Ag85A-PEGNio control at 4 µg/mL GAE significantly decreased intracellular Mtb viability, resulting in a 33.76% reduction in metabolic activity compared with the untreated control. Notably, treatment with Ag85A-PEGNio/EEP produced a dose-dependent inhibitory effect on intracellular Mtb, reducing bacterial metabolic activity by 23.01% and 51.19% at concentrations of 2 and 4 µg/mL GAE, respectively. Furthermore, the anti-mycobacterial activity of Ag85A-PEGNio/EEP at 4 µg/mL GAE was significantly greater than that observed with conventional isoniazid treatment. These findings demonstrate the enhanced efficacy of Ag85A-PEGNio/EEP against intracellular Mtb and highlight its potential as a targeted therapeutic strategy for tuberculosis.

### 2.3. Effect of Ag85A-PEGNio/EEP Activity on Macrophage Cytokines in Response to Mtb Infection

**Figure 3:**
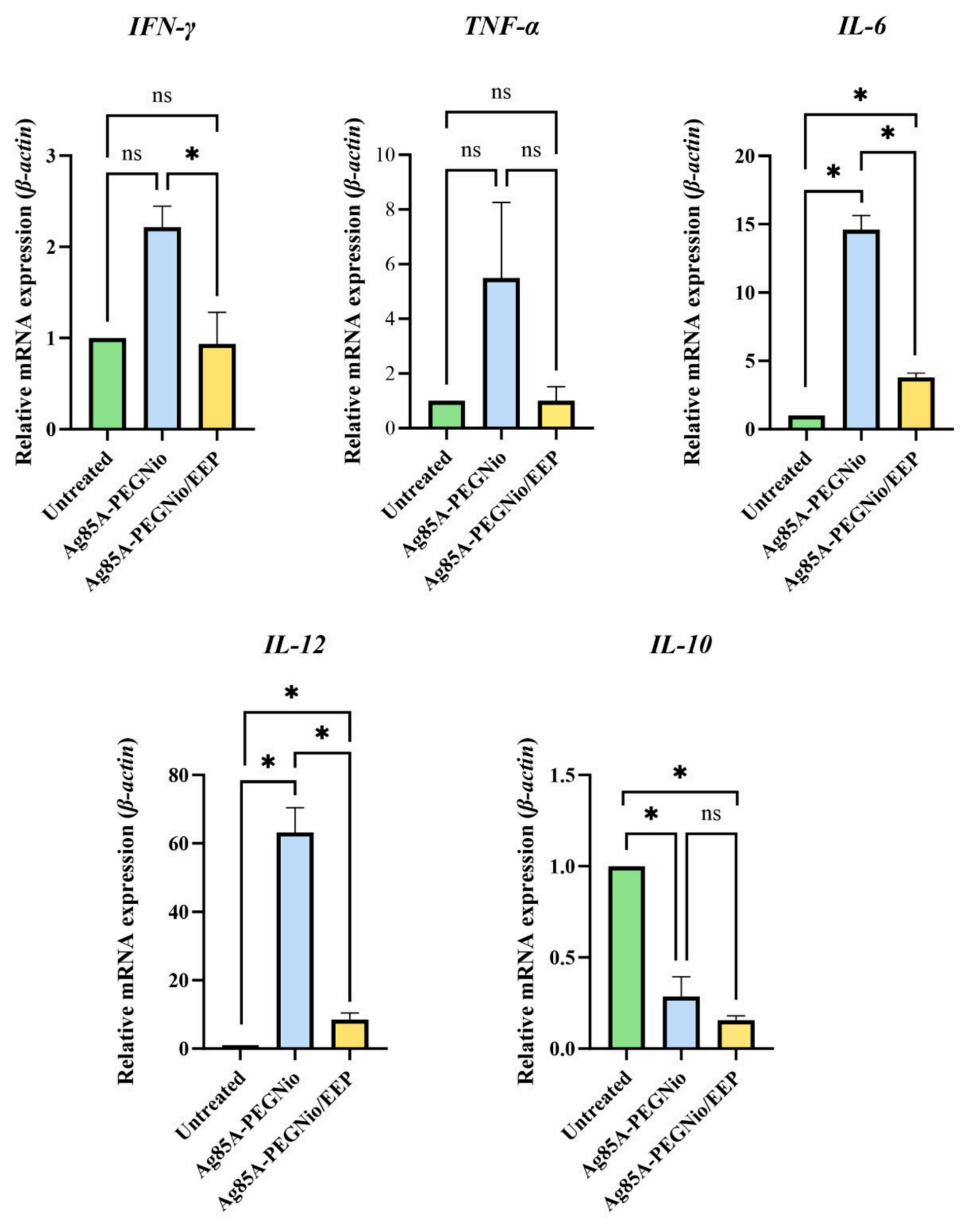
The relative expression level of macrophage cytokines in response to Mtb infection. Mtb-infected macrophages were treated with Ag85A-PEGNio/EEP for 5 days followed by RNA extraction. The mRNA expression of macrophage pro-inflammatory cytokines (*IFN-γ*, *TNF-α*, *IL-12*, and *IL-6*) and anti-inflammatory cytokine (*IL-10*) was determined by real-time qPCR. * *p* < 0.05 when compared to each group. ns: not significant when compared to each group.

The relative mRNA expression level of pro-inflammatory cytokines (*IFN-γ*, *TNF-α*, *IL-12* and *IL-6*) and anti-inflammatory cytokine (*IL-10*) in Mtb-infected macrophages was quantified by real-time qPCR. As shown in Figure 3, treatment with Ag85A-PEGNio/EEP significantly increased the expression of *IL-12* and *IL-6* by 8.4-fold and 3.8-fold, respectively, compared with untreated infected controls. In contrast, *IL-10* expression was significantly downregulated, showing a 6.4-fold reduction following Ag85A-PEGNio/EEP treatment. No significant changes were observed in the expression levels of *IFN-γ* and *TNF-α* relative to the untreated control group. Treatment with Ag85A-PEGNio demonstrated the significant upregulation of *IFN-γ* (2.2-fold), *TNF-α* (5.5-fold), *IL-12* (63.2-fold), and *IL-6* (14.6-fold) compared with the untreated infected control. Notably, although Ag85A-PEGNio induced robust expression of pro-inflammatory cytokines, encapsulation of EEP within the niosomal formulation attenuated this response. The lower expression levels of *IFN-γ*, *TNF-α*, *IL-12*, and *IL-6* observed in the Ag85A-PEGNio/EEP-treated group suggest a modulatory effect exerted by EEP on macrophage inflammatory responses. These findings indicate that Ag85A-PEGNio/EEP may promote a balanced immune response during Mtb infection by enhancing the expression of key host-protective cytokines, particularly *IL-12* and *IL-6*, while suppressing the expression of anti-inflammatory cytokine *IL-10*. The attenuation of excessive pro-inflammatory cytokine production further highlights the immunomodulatory and anti-inflammatory properties of the encapsulated EEP.

### 2.4. Proteomics Profiles of Mtb-Infected Macrophage

**Figure 4:**
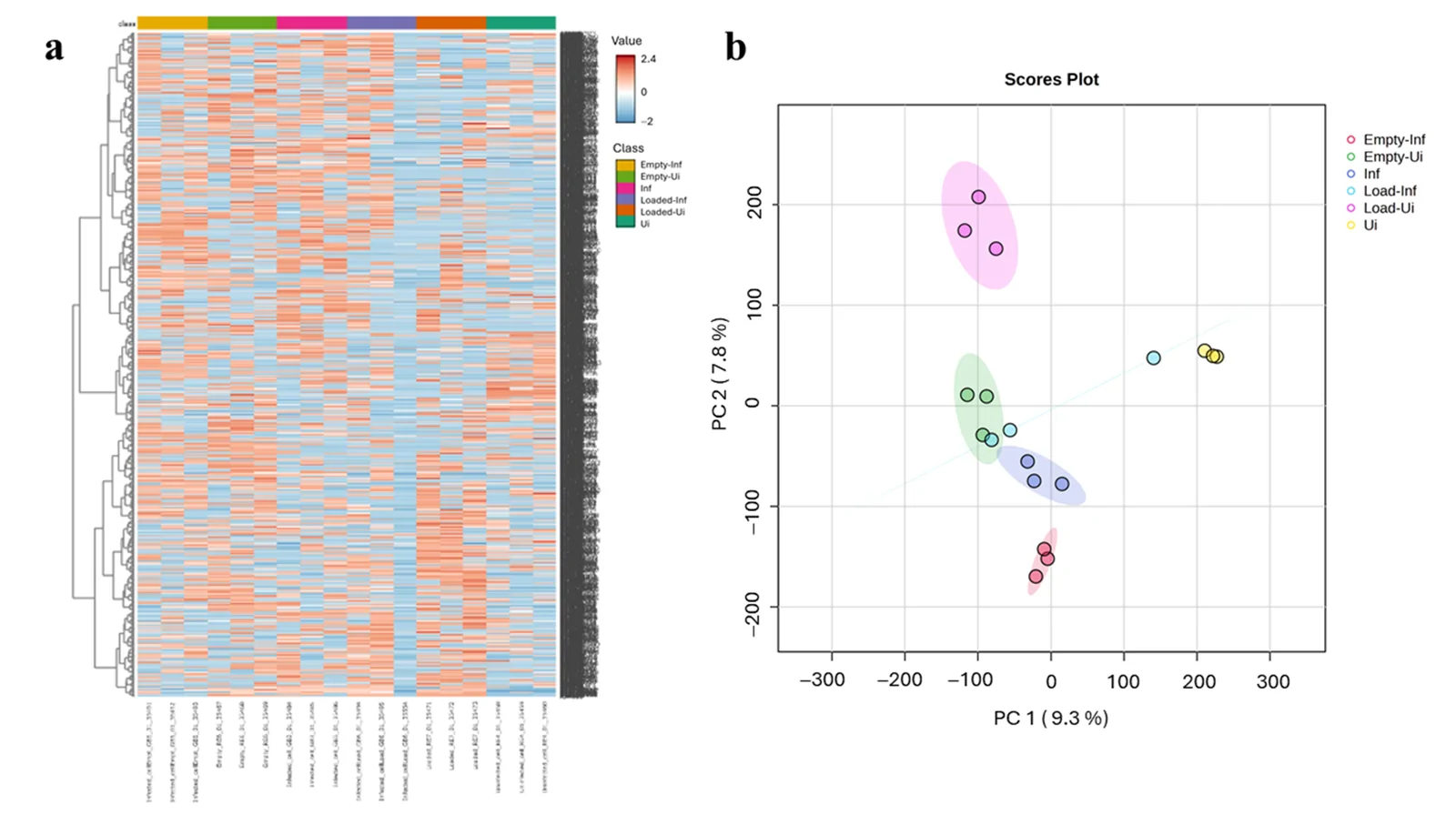
Proteome profiling studies of Mtb-infected macrophage following Ag85A-PEGNio/EEP treatment. Heatmap visualization (**a**) and Principal component analysis (**b**) of global proteomic profiles of Mtb-infected macrophages following Ag85A-PEGNio/EEP treatment for 5 days. Each data point represents one technical replicate, with three technical replicates per group. Ellipses represent 95% confidence intervals for each experimental group. Experimental groups are as follows: Ag85A-PEGNio/EEP-treated Mtb-infected macrophages (LoadInf); Ag85A-PEGNio/EEP-treated uninfected macrophages (LoadUi); empty niosome-treated Mtb-infected macrophages (EmpInf); empty niosome-treated uninfected macrophages (EmpUi); untreated Mtb-infected macrophages (Inf) and untreated uninfected macrophages (Ui).

To investigate alterations in the proteomic profile of Mtb-infected macrophages, label-free quantitative proteomics was performed. A total of 7313 proteins were identified across all samples by LC-MS/MS analysis. Prior to downstream analysis, principal component analysis (PCA) was applied to assess the uniformity and technical reproducibility of protein abundance levels across replicates. The PCA scores plot revealed clear and distinct separation among all experimental groups: Ag85A-PEGNio/EEP-treated infected versus uninfected macrophages (LoadInf vs. LoadUi), empty niosome-treated infected versus uninfected macrophages (EmpInf vs. EmpUi), and untreated Mtb-infected versus uninfected macrophages (Inf vs. Ui). PC1 and PC2 accounted for 9.3% and 7.8% of the total variance, respectively. Each group formed well-defined clusters within 95% confidence ellipses, with tight grouping of the three technical replicates per group, confirming good technical reproducibility. The distinct separation observed along PC1 reflects the combined influence of Mtb infection status and Ag85A-PEGNio/EEP treatment on the macrophage proteome, suggesting that these conditions drive fundamentally distinct proteomic responses (Figure 4).

**Figure 5:**
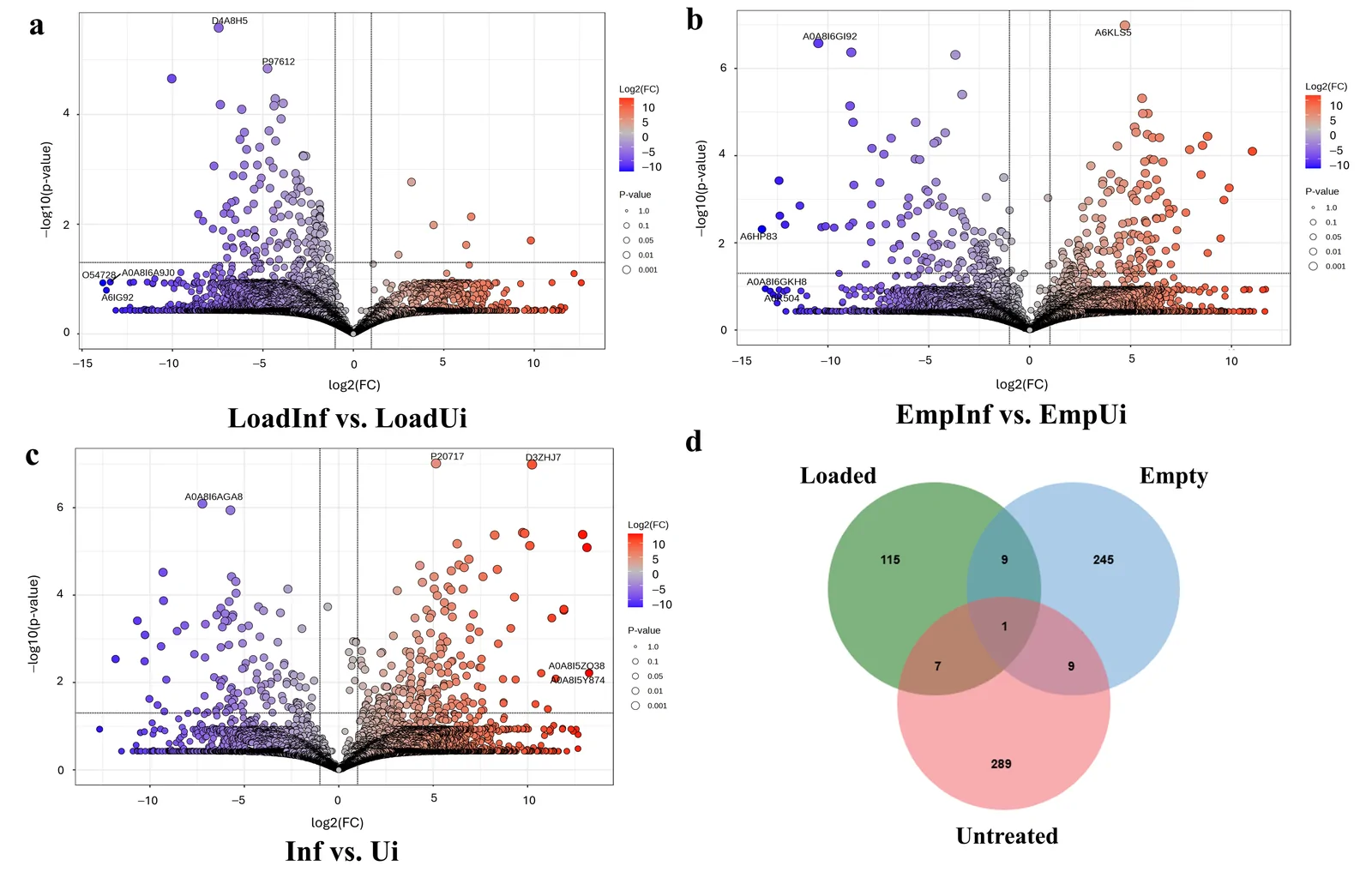
Differential proteomic analysis of Mtb-infected macrophages following Ag85A-PEGNio/EEP treatment for 5 days. Volcano plots illustrating significantly altered protein abundance in (**a**) Ag85A-PEGNio/EEP-treated Mtb-infected macrophages versus Ag85A-PEGNio/EEP-treated uninfected macrophages (LoadInf vs. LoadUi), (**b**) empty niosome-treated Mtb-infected macrophages versus empty niosome-treated uninfected macrophages (EmpInf vs. EmpUi), and (**c**) untreated Mtb-infected macrophages versus untreated uninfected macrophages (Inf vs. Ui). In each volcano plot, the x-axis represents the log_2_ fold change and the y-axis represents the −log_10_ *p*-value. Proteins meeting the significance threshold of log_2_ fold change > 1 and *p* < 0.05 are considered differentially expressed; red and blue dots denote significantly upregulated and downregulated proteins in the infected or treated condition relative to the respective uninfected control, respectively. Grey dots represent proteins that did not meet the significance threshold. (**d**) Venn diagram depicting the overlap and unique distribution of differentially expressed proteins (both upregulated and downregulated combined) across all three comparison groups.

The proteomes of Mtb-infected cells in different treatments were comprehensively analyzed to identify the differentially expressed proteins (DEPs; >log2FC, *p*-value < 0.05) by visualizing volcano plots (Figure 5a–c). For this, three analysis groups were designed, namely, Mtb-infected cells compared to uninfected control following Ag85A-PEGNio/EEP treatment (LoadInf vs. LoadUi), Mtb-infected cells compared to uninfected control following empty-niosome treatment (EmpInf vs. EmpUi) and Mtb-infected cells compared to uninfected cells (Inf vs. Ui). The Volcano plot analysis revealed the following: 132 DEPs (6 upregulated, 126 downregulated) in LoadInf vs. LoadUi (Figure 5a), 264 DEPs (150 upregulated, 114 downregulated) in EmpInf vs. EmpUi (Figure 5b), and 306 DEPs (193 upregulated, 113 downregulated) in Inf vs. Ui (Figure 5c). The distribution of DEPs across different experimental groups was visualized by Venn diagram-based distribution analysis to compare these datasets. We found that a total 115 unique proteins were identified in Mtb-infected cells after Ag85A-PEGNio/EEP treatment (Figure 5d).

### 2.5. Gene Ontology (GO) Enrichment and Pathway Analysis of Macrophages Responses to Mtb Infection Under Ag85A-PEGNio/EEP Treatment

**Figure 6:**
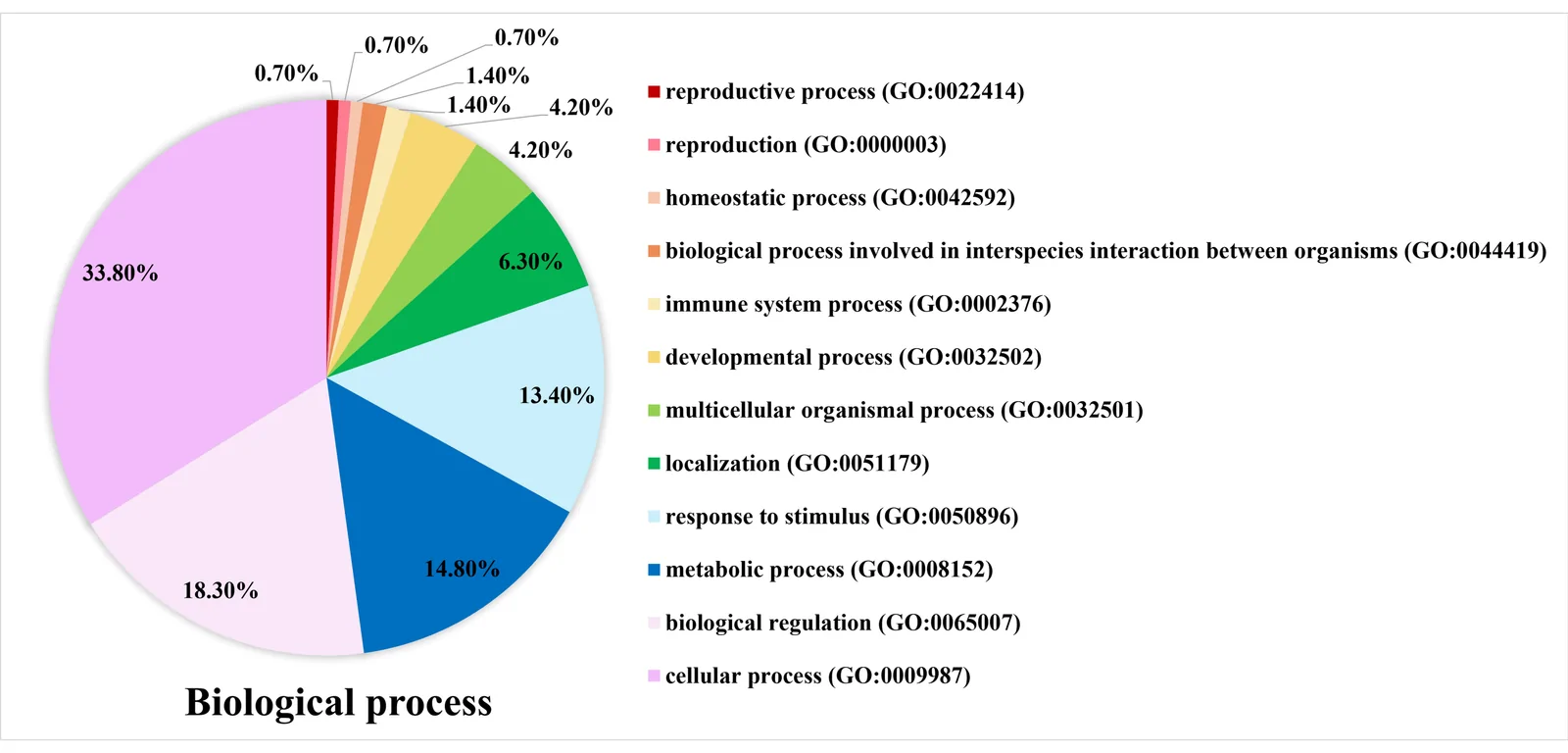
Pie charts illustrate the functional classification of unique proteins identified in Ag85A-PEGNio/EEP-treated Mtb-infected macrophages across biological processes via Protein ANalysis THrough Evolutionary Relationships (PANTHER).

To characterize the biological responses of Mtb-infected macrophage following Ag85A-PEGNio/EEP treatment, GO enrichment analysis was performed using the Protein ANalysis THrough Evolutionary Relationships (PANTHER) database [14]. The 115 unique proteins identified in Ag85A-PEGNio/EEP-treated Mtb-infected macrophages, as derived from the Venn diagram analysis, were subjected to classification based on biological process ontology. As shown in Figure 6, these proteins were represented across 12 pathways related to the macrophage response to infection, including homeostatic process (GO:0042592), immune system process (GO:0002376), response to stimulus (GO:0050896), metabolic process (GO:0008152), biological regulation (GO:0065007), and cellular process (GO:0009987).

According to our hypothesis, we specifically focused on proteins involved in macrophage immune system processes and response to stimulus. Twenty-three significant proteins were identified. Of 23 identified proteins, 22 proteins exhibited downregulation, except for the ATP-dependent DNA helicase (A0A0G2K5S2), which showed upregulation following Ag85A-PEGNio/EEP treatment. The assessment of these proteins and their biological processes and molecular function is listed in Table 1.

**Figure 7:**
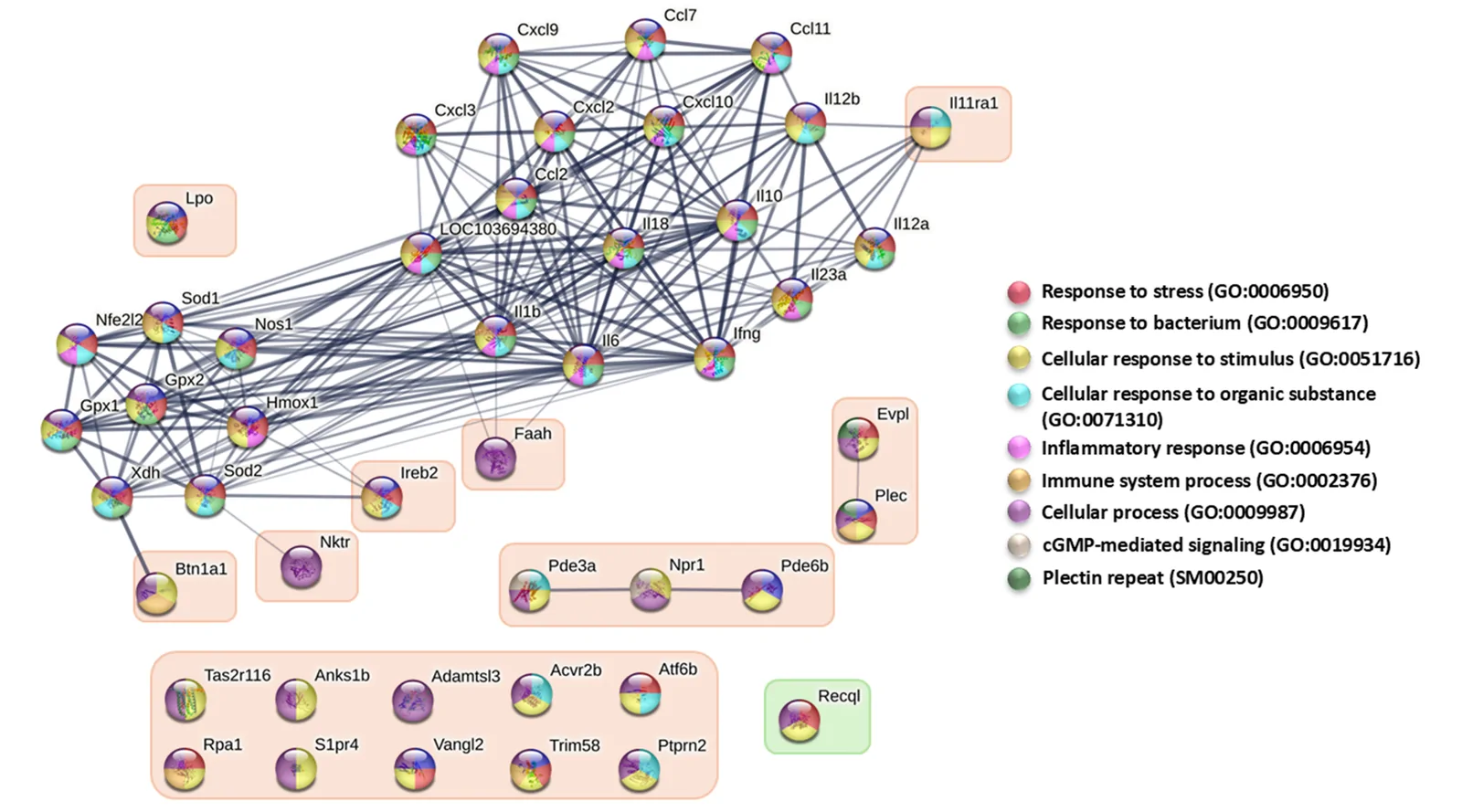
STRING protein–protein interaction (PPI) network of twenty-three significant proteins identified in Mtb-infected macrophages following Ag85A-PEGNio/EEP treatment. Nodes represent individual proteins, and edges indicate protein–protein interactions, with line thickness proportional to confidence scores, as shown in Table S2. Node colors represent the biological pathway of individual proteins. The abundance of downregulated and upregulated significant proteins are enclosed in the orange and green boxes, respectively.

The twenty-three significant proteins identified following Ag85A-PEGNio/EEP were analyzed for their protein–protein interaction (PPI) via STRING network analysis. As shown in Figure 7, Ag85A-PEGNio/EEP play a role in the alteration of various host responses to Mtb infection. The PPI network demonstrated the notable associations among the immune system process, detoxification of reactive oxygen species, and heme catabolic process. The network exhibited the interaction of Btn1a1, Nktr, and Ireb2 with Sod1/2, Gpx1/2, and Hmox1, indicating their expression involved in the antioxidant system via the Nfe2l2 (Nrf2) pathway. Moreover, the interaction of Il11ra and Faah was found to be involved in the immune system process via the cytokine and chemokine activity pathway, as they are associated with Il12a, Il12b, Il10, Il6, Il23a, Il1b, Infg, and tnfa. In addition, three proteins including Pde3a, Npr1, and Pde6b were identified via the cGMP signaling pathway. These results suggest that Ag85A-PEGNio/EEP play a role in the alteration of various host responses to Mtb infection.

## 3. Discussion

In this study, we demonstrated that Ag85A-PEGNio/EEP nanoparticles were efficiently internalized by macrophages and exhibited notable anti-mycobacterial activity against intracellular Mtb. These findings support our hypothesis that targeted nanodelivery systems can enhance both drug delivery into host cells and antimicrobial efficacy against intracellular pathogens.

Efficient uptake of Ag85A-PEGNio/EEP by macrophages is particularly important, as Mtb primarily resides and replicates within these host cells [2]. Following internalization, the niosomes trafficked to the host phagolysosome, facilitating the targeted delivery and release of encapsulated EEP directly to the site of infection. These findings are consistent with previous studies demonstrating that niosomes improve intracellular drug accumulation and enhance therapeutic outcomes in tuberculosis models [15]. Furthermore, CLSM analysis revealed close spatial localization of Ag85A-PEGNio/EEP with intracellular Mtb within infected macrophages, suggesting that the functionalization with the Ag85A aptamer facilitates more specific targeting toward Mtb-infected cells. This high binding affinity of the Ag85A aptamer for Mtb antigens likely contributes to this targeting capability, providing a distinct advantage over conventional non-targeted formulations [13].

Evaluation of intracellular antimycobacterial activity revealed that treatment with INH did not significantly reduce the metabolic activity of intracellular Mtb compared with untreated controls. This result is consistent with previous reports demonstrating that although exhibits potent activity against extracellular Mtb, its efficacy is substantially reduced within infected macrophages, resulting in less than a 0.5-log_10_ CFU/mL reduction in intracellular Mtb in J774A.1 macrophages [16]. The intracellular localization of Mtb within macrophages is known to influence both bacterial persistence and drug susceptibility. Specifically, Mtb residing in proximity to the nuclear region often exhibits reduced replication rates and enhanced drug tolerance, whereas bacteria located in peripheral regions of cell tend to be more metabolically active and susceptible to antimicrobial treatment [17]. In this study, CLSM analysis confirmed that intracellular Mtb localized within lysosomal compartments in the perinuclear region of infected macrophages. Notably, Ag85A-PEGNio/EEP was also trafficked to these compartments, enabling localized delivery of EEP directly to intracellular Mtb. This targeted intracellular distribution may explain the significantly superior intracellular antimycobacterial efficacy of Ag85A-PEGNio/EEP compared with INH.

Although REMA provides a rapid and reproducible assessment of mycobacterial metabolic activity, it does not directly quantify viable bacterial burden. Therefore, intracellular CFU enumeration remains necessary to confirm bactericidal efficacy. Future studies should incorporate CFU assays to further validate the intracellular antimycobacterial activity of Ag85A-PEGNio/EEP.

The investigated intracellular activity is consistent with our previous findings demonstrating the in vitro antimycobacterial efficacy of Ag85A-PEGNio/EEP following 7 days of treatment [13]. The antimicrobial mechanisms of EEP against *Mycobacterium* spp. have been described to involve various biological effects, including disruption of cell membrane integrity, inhibition of nucleic acid synthesis, and interference with bacterial energy metabolism [10,18]. Nevertheless, it is important to consider that the phagocytic uptake of high amounts of nanoparticles may induce a state of particle overload within macrophages. Excessive nanoparticle accumulation has been reported to activate host defense pathways, including autophagy, oxidative stress responses, and lysosomal signaling, which may subsequently restrict the intracellular pathogen replication [19]. Therefore, the potential influence of nanoparticle burden on host cell responses should be carefully considered when interpreting the anti-mycobacterial effects observed in this study.

Unlike INH, which primarily acts through inhibition of mycolic acid biosynthesis, EEP exhibits both direct anti-mycobacterial activity and immunomodulatory properties, including regulation of inflammatory cytokine responses. Therefore, the present study focused on evaluating EEP as a host-directed therapeutic agent rather than as a drug delivery system for conventional anti-tuberculosis agents. Nevertheless, an important consideration for future development of the Ag85A-PEGNio/EEP is its application as a targeted delivery system. The potential of the Ag85A-targeted niosomal platform for the delivery of conventional anti-tuberculosis drugs, such as INH, requires further exploration. Future studies comparing Ag85A-PEGNio/EEP, Ag85A-PEGNio/INH, and Ag85A-PEGNio/EEP + INH may provide valuable insights into the relative contributions of direct anti-mycobacterium activity and host-directed immunomodulation.

Analysis of cytokine gene expression demonstrated that Ag85A-PEGNio treatment significantly increased the expression of *IFN-γ*, *TNF-α*, *IL-12* and *IL-6* in Mtb-infected NR8383 macrophages. This result may be associated with cholesterol-mediated activation of inflammatory signaling pathways, including IκB kinase/NF-κB and mitogen-activated protein kinases (MAPK) cascades, such as MKK3/p38, Erk1/2, and JNK1/2 [20,21]. In contrast, treatment with Ag85A-PEGNio/EEP did not significantly alter *IFN-γ* or *TNF-α* expression relative to untreated control. While the expression levels of *IL-12* and *IL-6* were significantly higher than those observed in untreated control. The encapsulated EEP might modulate excessive inflammatory responses while preserving key host defense mechanisms. Consistently, Consistently, previous studies have suggested that the anti-inflammatory activity of EEP is largely attributed to its ability to suppress NF-κB and MAPK signaling pathways, reduce ROS generation, and inhibit pro-inflammatory mediator release [22]. Importantly, Ag85A-PEGNio/EEP treatment significantly reduced the expression of *IL-10*, an immunosuppressive cytokine that is frequently induced during infection with virulent Mtb strains. Elevated *IL-10* levels have been associated with impaired phagosome-lysosome fusion, reduced apoptosis of infected macrophages, and enhanced bacterial survival within host cells [23]. Therefore, suppression of *IL-10* expression by Ag85A-PEGNio/EEP may facilitate phagosome maturation and improve intracellular bacterial clearance [24]. Although these pathways were not directly investigated in the present study, they may contribute to the immunomodulatory effects of Ag85A-PEGNio/EEP and warrant further mechanistic investigation. Taken together, these transcriptional changes suggest that Ag85A-PEGNio/EEP not only enhances intracellular bacterial clearance but also modulates host immune responses in a manner.

Proteomics analysis revealed that treatment with Ag85A-PEGNio/EEP resulted in marked alterations in the protein expression profiles of Mtb-infected macrophages. Functional classification of the 115 proteins uniquely identified in Ag85A-PEGNio/EEP-treated Mtb-infected macrophages indicated modulation of pathways associated with macrophage responses to infection. The majority of proteins involved in response to stimulus and immune system processes were downregulated following Ag85A-PEGNio/EEP treatment relative to the uninfected control, including Btn1a1, Il11ra1, S1pr4, Trim58, Vangl2, and Atf6b. The downregulation of these proteins is of particular significance given their well-established roles in immune modulation and inflammatory signaling. Btn1a1 has been reported to suppress T-cell activity through direct binding to the T-cell receptor (TCR), thereby inhibiting lymphocyte proliferation and cytokine secretion [25]. Il11ra1 encodes the alpha subunit of the interleukin-11 (IL-11) receptor, a cytokine that is upregulated in the human lung in response to infection [26]. Consistent with this, blockade of IL-11 signaling has been shown to reduce histopathological damage and neutrophilic infiltration in lung tissue of genetically susceptible mice infected with Mtb [27]. Collectively, these observations suggest that Ag85A-PEGNio/EEP may moderate IL-11 signaling pathways to attenuate macrophage-mediated inflammation and cell death.

The downregulation of additional proteins further suggests interference with pro-inflammatory signaling and cytokine release. S1pr4 encodes a receptor for bioactive sphingosine metabolites predominantly expressed in macrophages. S1PR4 has been implicated in oxylipin-mediated signaling through co-engagement with formyl peptide receptor 2, promoting JNK phosphorylation and macrophage M1 polarization, which drives the secretion of pro-inflammatory cytokines and contributes to pulmonary inflammatory infiltration [28]. Trim58 has been identified as a negative regulator of innate immune signaling through promotion of TLR2 degradation; loss of Trim58 function in myeloid cells results in constitutive TLR2 upregulation and exaggerated production of IL-1β in response to pro-inflammatory stimuli, including TNF-α and IFN-γ [29]. Similarly, Vangl2 has been characterized as a negative regulator of NF-κB signaling through modulation of p65 protein stability, thereby suppressing the transcription of pro-inflammatory cytokines [30]. Downregulation of Atf6b has likewise been associated with attenuation of inflammatory responses and reduced cytokine expression [31]. Taken together, these findings suggest that Ag85A-PEGNio/EEP may moderate cytokine-driven inflammatory cascades, thereby reducing the risk of excessive pulmonary inflammation during Mtb infection.

Analysis of the STRING protein–protein interaction (PPI) network revealed a multifaceted host response characterized by the coordinated modulation of pro-inflammatory signaling and antioxidant defense mechanisms. Ag85A-PEGNio/EEP treatment was associated with activation of the Nfe2l2 (Nrf2)-mediated antioxidant pathway, as evidenced by interaction networks involving Sod1, Sod2, Gpx1, Gpx2, and Hmox1. The Nrf2 pathway is a master regulator of cellular redox homeostasis and has been previously shown to confer protection against oxidative stress induced by mycobacterial infection [32,33]. Additionally, interaction of Nktr within this cluster suggests that macrophage innate immune signaling may be primed for amplification through natural killer (NK) cell recruitment. Nktr has been reported to enhance macrophage-mediated signaling for NK cell recruitment, thereby amplifying pro-inflammatory responses and promoting robust microbicidal ROS production [34]. This coordinated antioxidant response is likely attributable to the flavonoid constituents of the EEP component within the niosomal delivery system, which may moderate oxidative damage typically induced by the mycobacterial respiratory burst.

Three proteins associated with the cGMP-mediated signaling pathway were additionally identified following Ag85A-PEGNio/EEP treatment, namely Pde3a, Npr1, and Pde6b. This observation may reflect interference with host cGMP signaling, a mechanism previously reported to be exploited by Mtb through the direct secretion of bacterially derived cyclic AMP (cAMP) into host cells [35]. Perturbation of this signaling axis has been shown to trigger the production of type I interferons (IFNs) and pro-inflammatory cytokines in host cells [36], suggesting that Ag85A-PEGNio/EEP may moderate this pathway to limit Mtb-driven immune evasion.

Furthermore, modulation of Ireb2 expression suggests that Ag85A-PEGNio/EEP treatment may influence intracellular iron homeostasis within the macrophage. Given that iron is an essential nutrient for Mtb survival and replication, downregulation of Ireb2 which is a key regulator of iron uptake and storage, may serve to restrict the availability of intracellular iron, thereby limiting bacterial replication [37,38]. Collectively, these findings suggest that Ag85A-PEGNio/EEP coordinates a multilayered defense response within the macrophage, reprogramming it from a passive nutrient reservoir into an active metabolic effector that restricts Mtb survival through simultaneous modulation of inflammatory signaling, oxidative defense, and iron sequestration.

A limitation of this study is that the proteomic findings were not validated using antibody-based methods such as Western blot or ELISA. Although such approaches are commonly used to corroborate mass spectrometry-based results, they carry recognized limitations that constrain their utility as a validation standard, including variable antibody specificity, cross-reactivity, and the limited availability of highly specific antibodies for many target proteins [39,40,41]. Differences in sample preparation between MS-based proteomics and immunoassay platforms can further contribute to discrepancies that reflect methodological divergence rather than inaccuracy of the proteomic data. In the context of the present study, well-validated antibodies suitable for quantitative Western blot or ELISA were not available for several of the candidate proteins identified, and developing custom antibodies with adequate specificity would have required resources and time beyond the scope of this work.

In place of antibody-based validation, the biological plausibility of the identified proteins was assessed through protein–protein interaction (PPI) analysis using the STRING database [42], which integrates evidence from experiments, curated databases, and literature to predict functional associations between proteins. This analysis revealed direct associations between significant proteins identified following Ag85A-PEGNio/EEP treatment and immune-related and antioxidant pathways, including clustering of Il11ra and Faah with cytokine/chemokine-related proteins (Il12a, Il12b, Il10, Il6, Il23a, Il1b, Ifng, and Tnf), clustering of Btn1a1, Nktr, and Ireb2 with antioxidant system components (Sod1/2, Gpx1/2, and Hmox1), and association of Pde3a, Npr1, and Pde6b with the cGMP signaling pathway. These bioinformatic associations were further corroborated by independent functional assays, including intracellular antimycobacterial activity and cytokine expression analyses, which provided experiment-based evidence consistent with the proteomic observations. We therefore consider the combination of bioinformatic PPI analysis and functional validation to be a reasonable and complementary alternative to antibody-based methods in the context of this study. Future work may incorporate targeted MS-based approaches, such as parallel reaction monitoring or synthetic peptide standards, for antibody-independent, protein-level quantification of these targets.

Although the present study demonstrates the promising therapeutic potential of Ag85A-PEGNio/EEP in an in vitro macrophage model, several challenges must be addressed before clinical translation can be realized. One of the major obstacles in tuberculosis therapy is the efficient delivery of therapeutic agents to intracellular Mtb residing within macrophages, as drugs must traverse multiple biological barriers, including the plasma membrane and phagosomal membrane, to reach the site of infection. In the present study, Ag85A-PEGNio/EEP demonstrated efficient macrophage internalization and intracellular Mtb targeting in vitro, indicating its potential to enhance drug accumulation at infected sites. Furthermore, the Ag85A-targeted nanodelivery platform may reduce dose-related toxicity through selective targeting of Mtb. Nevertheless, these potential advantages require validation through comprehensive in vivo biodistribution and pharmacokinetic studies. Therefore, future studies employing advanced three-dimensional granuloma models and animal model of tuberculosis are warranted to evaluate therapeutic efficacy, pulmonary targeting, biosafety, and pharmacokinetic behavior before clinical translation.

In summary, Ag85A-PEGNio/EEP demonstrated effective localization with Mtb that resides within infected macrophages and exhibited anti-mycobacterial activity. It also regulates macrophage responses through increasing the production of pro-inflammatory cytokines while concurrently suppressing the anti-inflammatory cytokine. Ag85A-PEGNio/EEP treatment probably modulates multiple immune-regulatory proteins and mitigates host oxidative damage, which is typically induced by the mycobacterial respiratory burst.

## 4. Materials and Methods

### 4.1. Materials

Propolis powder was kindly provided by Bee Product Industry (Lamphun, Thailand). Ag85A-PEGNio/EEP were fabricated and characterized and the total phenolic content (TPC) was quantified by the Folin–Ciocalteu method as previously reported [13]. The physicochemical characteristics of Ag85A-PEGNio/EEP used in this study are presented in Table S1. Nile-red dye was purchased from Sigma-Aldrich (St. Louis, MO, USA). Ag85A Apt (5′-GCT GTG TGA CTC CTG CAA GCG GGA AGA GGG TAA GGG GAG GGA GGG TAA CGC GGA GAA GGC AAG CAG CTG TAT CTT GTC TCC-3′), according to Ansari et al. [43], was synthesized and modified with amine and Cy5 at 3′ and 5′ ends, respectively, by Integrated DNA Technologies (Coralville, IA, USA). Mouse anti- CD41 monoclonal antibody was purchased from Thermo Fisher Scientific (Waltham, MA, USA). FITC-conjugated rabbit anti-mouse IgG antibody was kindly gifted by Assoc. Prof. Dr. Sawitree Chiampanichayakul, Department of Medical Technology, Faculty of Associated Medical Sciences, and Chiang Mai University. The cells studied in this article are purchased from Biomedia (Bangkok, Thailand) Co., Ltd. The ATCC number is CRL-2192 (NR8383: Rat alveolar macrophage).

### 4.2. Mycobacterial Strain and Culture

Mtb H37Rv was cultured in Middlebrook 7H9 broth supplemented with 10% oleic albumin dextrose catalase (OADC) (Becton Dickinson, Franklin Lakes, NJ, USA) at 37 °C for 14 days. Then, the bacilli were adjusted to 0.5 McFarland standards containing approximately 1 × 10^7^ CFU/mL for further experiments. This work was approved by the Institutional Biosafety Committee, Chiang Mai University (approval no. CMUIBC-0565004).

### 4.3. Cell Culture

Alveolar macrophage cells (NR8383) were cultured in Ham’s F-12K (Kaighn’s) medium supplemented with 15% fetal bovine serum (FBS) penicillin (100 units/mL) and streptomycin (100 µg/mL). The cell line will be maintained in a humidified atmosphere of 5% CO_2_ at 37 °C.

### 4.4. Intracellular Uptake of Ag85A-PEGNio/EEP in Macrophage

The NR8383 cells (5 × 10^5^ cells/well) were cultured in 8-well culture slides for 24 h. The Ag85A-PEGNio/EEP was stained with 20 µg/mL of Nile red (NR) solution for 20 min at room temperature followed by washing with PBS [44]. The NR-labeled PEGNio/EEP were added into the cells and incubated for 2 h at 37 °C. After incubation, the cells were fixed with 4% paraformaldehyde followed by adding 2% bovine serum albumin (BSA) for 30 min. For NR8383 plasma membrane visualization, the mouse anti-CD41 monoclonal antibody was added and incubated with FITC-conjugated rabbit anti-mouse polyclonal antibody. After 30 min, the nuclei were counterstained by DAPI. Intracellular uptake of the niosomes in macrophages was observed, and images were acquired using a Confocal Laser Scanning Microscope (CLSM).

For quantitative measurement, the intracellular uptake was analyzed by flow cytometry. NR8383 cells were seeded in 24-well tissue culture plate and incubated with NR-labeled PEGNio/EEP. After incubation, the cells were harvested and washed with FACS diluent. Then, the pellet was adjusted to a final amount of 1 × 10^5^ cells and incubated with mouse anti-CD41 antibody for 30 min. After a washing step, the FITC-conjugated anti-mouse antibody was added for 30 min, followed by washing. The samples were resuspended in 1% paraformaldehyde and subjected to a flow cytometer (BD Accuri™ C6 flow cytometer; BD Biosciences, San Jose, CA, USA).

### 4.5. Localization of Ag85A-PEGNio/EEP and Mtb in Mtb-Infected Macrophages

Localization of Ag85A-PEGNio/EEP in the macrophage was investigated. The NR8383 cells were grown in an 8-well culture slide for 24 h. The Mtb cell wall was stained with Auramine O for 15 min. Then, the macrophages were infected with 10 MOI of Auramine O-stained Mtb for 18 h. The PEGNio/EEP was conjugated with a Cy5-modified Ag85A aptamer via an EDC/NHS reaction [13] and incubated with the Mtb-infected macrophages for 2 h. The cells were stained with LysoTracker Red and counterstained with DAPI for phagolysosomes and nuclei, respectively, followed by the anti-fade reagent. The localization of Mtb, nanoparticles, and phagolysosomes was visualized and analyzed using CLSM.

### 4.6. Intracellular Metabolic Activity of Mtb 

The macrophages (5 × 10^5^ cells/well) were cultured in a 96-well plate for 24 h. The cells were infected with 10 MOI of Mtb for 18 h. After incubation, the cells were washed with PBS to remove extracellular bacteria. Then, the Mtb-infected macrophages were treated with various concentrations of TPC contained in Ag85A-PEGNio/EEP and incubated at 37 °C for 7 days. The cells were lysed with sterile deionized (DI) water and incubated with 0.01% resazurin for 24 h [45]. The intracellular Mtb viability was determined and quantified by mean fluorescence intensity (MFI) at 530–560/590 nm. The percentage of intracellular Mtb viability was calculated using the equation as follows:

$$\%\mathrm{Viability}\;\mathrm{of}\;\mathrm{intracellular}\;\mathrm{Mtb}\;=\frac{\mathrm{MFI}\;\mathrm{of}\;\mathrm{test}-\mathrm{MFI}\;\mathrm{of}\;\mathrm{blank}\;}{\mathrm{MFI}\;\mathrm{of}\;\mathrm{growth}\;\mathrm{control}}\;\times 100$$

In this equation, the MFI of the test was the MFI of the intracellular Mtb treated with Ag85A-PEGNio/EEP.

MFI of blank was the MFI of the niosome control.

MFI of growth control was the MFI of untreated intracellular Mtb.

### 4.7. Effect of Ag85A-PEGNio/EEP on Cytokine Responses in Mtb-Infected NR8383 Macrophages

#### 4.7.1. Preparation and Treatment

NR8383 cells (2 × 10^6^ cells/well) were cultured in a 6-well plate for 24 h and infected with 10 MOI of Mtb. Then, the infected cells were treated with Ag85A-PEGNio/EEP and incubated at 37 °C for 5 days. INH-treated and untreated control conditions were performed for each assay. The treated cells were pelleted by centrifugation, and the supernatant was discarded before performing RNA extraction.

#### 4.7.2. RNA Extraction and cDNA Synthesis

Mtb-infected NR8383 treated with Ag85A-PEGNio/EEP were harvested, and the total RNA was isolated using a TRIzol Reagent (Invitrogen, Carlsbad, CA) according to the manufacturer’s instruction with some modifications. After adding TRIzol Reagent, the cell suspension was placed at −80 °C overnight. Following sorting into Trizol, the cell suspension was centrifuged, the supernatant (containing host RNA) was collected and transferred to a new microcentrifuge tube. Fresh Trizol and 0.1 mm sized zirconia-silica bead was added to the microcentrifuge tube containing cell pellet (bacterial debris). Mycobacterial cells were bead beating six times (speed level 1, 45 s) by OMNI Bead Ruptor (USA Scientific, Inc., Ocala, FL, USA) [46].Then, the part of Trizol containing the host cell RNA was re-added into the same tube to perform RNA extraction, according to Pisu et al. [47]. Total RNA was quantified by measuring the absorbance at 260/280 nm. The RNA was reverse transcribed into cDNA using FastKing gDNA Dispelling RT SuperMix (TIANGEN, Beijing, China).

#### 4.7.3. The Relative mRNA Expression of Macrophage Cytokines

In this study, the specific primers of macrophage cytokines were designed as shown in Table 2. The amplifications with specific primers were quantified using quantitative Real-Time PCR (qRT-PCR). The PCR reaction mixture (20 μL) consists of the following components: 2× SensiFAST SYBR^®^ No-ROX Mix (Bioline Reagents Ltd., London, UK), 10 µM of each primer, and 250 ng of cDNA template. Real-time PCR assay was performed using The CFX96 Touch Real-Time PCR Detection System (Bio-Rad Laboratories Inc., Hercules, CA). PCR conditions are shown in Table 3. The expression of the target mRNA level was analyzed by the 2^−∆∆CT^ method and expressed as relative fold change when normalized with a housekeeping gene for macrophage (*β-actin*).

### 4.8. Proteomics Analysis 

#### 4.8.1. Sample Preparation

Proteomic analysis was performed to characterize the Mtb-infected macrophage proteomic response after treatment with Ag85A-PEGNio/EEP, following the protocol described by Sangboonruang et al. [48]. Briefly, macrophages (2 × 10^6^ cells/well) were infected with Mtb and incubated for 18 h. Following incubation, cells were washed with phosphate-buffered saline (PBS) to remove extracellular bacteria, after which Ag85A-PEGNio/EEP (4 µg/mL GAE of TPC) was added and incubated at 37 °C for 5 days. In parallel, an untreated control condition (growth control) was maintained, in which Mtb-infected macrophages received no treatment and were incubated under identical conditions for the same 5-day period. Then, infected cells were washed with PBS, scraped into sterile tubes, and solubilized in 0.5% (*w*/*v*) SDS for 5 min. Cell lysates were then sonicated at 37 °C for 30 min, after which two volumes of ice-cold acetone were added to precipitate proteins. The suspensions were incubated at −20 °C for 8 h and subsequently centrifuged at 8000× *g* for 30 min at 4 °C. The resulting protein pellets were air-dried and resuspended in sample storage buffer comprising 50 mM NaH_2_PO_4_, 5 mM DTT, 0.25 M NaCl, and a protease inhibitor cocktail. Total protein concentrations were quantified using the Lowry method [49], with bovine serum albumin (BSA) serving as the protein standard.

#### 4.8.2. Tryptic Digestion and LC-MS/MS Analysis

For each sample, 5 µg of protein was solubilized in 10 mM ammonium bicarbonate (AMBIC). Disulfide bonds were reduced with 5 mM dithiothreitol (DTT) in 10 mM AMBIC at 60 °C for 1 h, followed by alkylation of free sulfhydryl groups with 15 mM iodoacetamide (IAA) in 10 mM AMBIC at room temperature for 45 min in the dark. Proteins were then digested overnight at 37 °C with sequencing-grade trypsin at an enzyme-to-protein ratio of 1:20 (*w*/*w*). The resulting peptides were dried by vacuum centrifugation and resuspended in 0.1% (*v*/*v*) formic acid (FA) prior to LC-MS/MS analysis.

Peptide samples (100 ng) were injected onto an Acclaim PepMap RSLC C18 column (75 µm I.D. × 15 cm, 2 µm particle size, 100 Å pore size; Thermo Scientific, Manchester, UK) coupled to an Ultimate 3000 Nano/Capillary LC System (Thermo Scientific, Manchester, UK) interfaced with a hybrid quadrupole Q-TOF impact II™ mass spectrometer (Bruker Scientific LLC, Billerica, MA, USA). Mobile phase A consisted of 0.1% (*v*/*v*) FA in water, and mobile phase B consisted of 0.1% (*v*/*v*) FA in 80% (*v*/*v*) acetonitrile. Peptides were eluted using a linear gradient of 5–55% mobile phase B over 30 min at a constant flow rate of 0.30 µL/min. The column temperature was maintained at 60 °C throughout the run. Data were acquired in positive ion mode with a spray voltage of 1.6 kV and nitrogen as the drying gas at a flow rate of 50 L/h. The scan range was set from *m*/*z* 150 to 2200 Da. Peptide fragmentation was performed by collision-induced dissociation (CID) using nitrogen as the collision gas at a fixed collision energy of 10 eV. Each sample was analyzed in technical triplicate.

#### 4.8.3. Bioinformatics Analysis

Raw LC-MS/MS data were processed using MaxQuant version 2.5.0.0 with the integrated Andromeda search engine [50], searched against the Rattus norvegicus proteome database retrieved from UniProt [42]. Label-free quantification (LFQ) was enabled, and the Match Between Runs feature was activated to minimize missing values across samples. Protein identification was accepted at a false discovery rate (FDR) of 1% and a significance threshold of *p* < 0.05. Database searches were conducted with the following parameters: trypsin as the digestion enzyme with a maximum of two missed cleavages permitted; a main search mass tolerance of 0.6 Da; carbamidomethylation of cysteine as a fixed modification; and oxidation of methionine and N-terminal protein acetylation as variable modifications. Only peptides of a minimum length of seven amino acids and containing at least one unique peptide were considered for protein identification and subsequent quantitative analysis.

Statistical analyses and data visualization, including volcano plots and heatmaps, were performed using MetaboAnalyst version 6.0 [51], with a significance threshold of *p* < 0.05. Differentially expressed proteins were subjected to functional classification and pathway enrichment analysis using the PANTHER Classification System version 19.0 [14]. Overlapping and unique proteins across experimental groups were visualized using Venn diagrams generated with the jVenn online tool (https://jvenn.toulouse.inrae.fr/app/index.html, (accessed on 12 July 2026)) [52]. Protein–protein interaction networks were constructed and analyzed using the STRING database version 12.0 [53].

### 4.9. Statistical Analysis

All results were represented as mean ± standard error of the mean (SEM) values of three independent experiments in triplicate. A comparison between groups was determined using one-way analysis of variance (ANOVA). The statistical differences were considered significant of *p*-value less than 0.05. All calculations and visualizations were performed using GraphPad Prism version 9.0. (GraphPad Software Inc., San Diego, CA, USA).

## 5. Conclusions

Taken together, Ag85A-PEGNio/EEP possesses both anti-mycobacterial activity, and immunomodulatory properties, enabling enhanced intracellular bacterial control while maintaining immune homeostasis during Mtb infection. This dual mode of action highlights its potential as a promising therapeutic platform for the treatment of intracellular TB infections. Further investigations using advanced granuloma models and in vivo infection systems may be used to validate its therapeutic efficacy and elucidate the molecular mechanisms underlying its host-directed and antimicrobial activities.

## Figures and Tables

**Table 1:** List of significant proteins identified for biological processes and molecular function by UniProtKB.

Protein ID	Protein Name	Gene Name	Biological Process	Molecular Function
**Upregulation**				
A0A0G2K5S2	ATP-dependent DNA helicase	*Recql*	DNA recombination, DNA repair, DNA replication	3′-5′ DNA helicase activity, ATP binding, ATP hydrolysis activity, nucleic acid binding
**Downregulation**				
A6I3W5	Serine/threonine-protein kinase receptor	*Acvr2b*		ATP binding, metal ion binding, transmembrane receptor protein serine/threonine kinase activity
P0C6S7	Ankyrin repeat and sterile alpha motif domain-containing protein 1B	*Anks1b*	chemical synaptic transmission, postsynaptic, ephrin receptor signaling pathway, receptor localization to synapse	ephrin receptor binding
A0A8I5ZPJ5	Activating transcription factor 6 beta	*Atf6b*	endoplasmic reticulum unfolded protein response, negative regulation of ATF6-mediated unfolded protein response, positive regulation of transcription by RNA polymerase II, regulation of transcription by RNA polymerase II, response to endoplasmic reticulum stress	DNA-binding transcription factor activity, RNA polymerase II-specific, RNA polymerase II cis-regulatory region sequence-specific DNA binding, sequence-specific double-stranded DNA binding, transcription cis-regulatory region binding
A0A8I6GI96	Envoplakin	*Evpl*	intermediate filament cytoskeleton organization, regulation of antibacterial peptide production, wound healing	structural molecule activity
A0A8I6ARG9	Interleukin 11 receptor subunit alpha 1	*Il11ra1*		cytokine receptor activity
D4A400	Lactoperoxidase	*Lpo*	defense response to bacterium, response to oxidative stress	heme binding, lactoperoxidase activity, peroxidase activity, thiocyanate peroxidase activity
P18910	Atrial natriuretic peptide receptor 1	*Npr1*	cell surface receptor signaling pathway, cGMP biosynthetic process, cGMP-mediated signaling	ATP binding, GTP binding, guanylate cyclase activity, protein kinase activity
Q62865	cGMP-inhibited 3′,5′-cyclic phosphodiesterase 3A	*Pde3a*	apoptotic signaling pathway, cAMP-mediated signaling, cellular response to cGMP, cGMP-mediated signaling, negative regulation of adenylate cyclase-activating G protein-coupled receptor signaling pathway, negative regulation of apoptotic process, negative regulation of cAMP/PKA signal transduction,	3′,5′-cGMP-inhibited cyclic-nucleotide phosphodiesterase activity, metal ion binding
D3ZDI8	Phosphodiesterase	*Pde6b*	cAMP-mediated signaling,	3′,5′-cyclic-GMP phosphodiesterase activity, metal ion binding
Q6S399	Plectin (Plectin-1)	*Plec*	intermediate filament cytoskeleton organization	actin binding
Q63475	Receptor-type tyrosine-protein phosphatase N2	*Ptprn2*	insulin secretion involved in cellular response to glucose stimulus, lipid metabolic process, neurotransmitter secretion, regulation of secretion	protein tyrosine phosphatase activity
Q7TP21	Replication protein A subunit	*Rpa1*	chromosome organization, DNA damage response, DNA repair, DNA replication, hemopoiesis	chromatin binding, chromatin-protein adaptor activity, damaged DNA binding, single-stranded DNA binding, DNA binding, zinc ion binding
D4AEH8	Sphingosine 1-phosphate receptor 4	*S1pr4*	adenylate cyclase-activating G protein-coupled receptor signaling pathway, G protein-coupled receptor signaling pathway, regulation of metabolic process	G protein-coupled receptor activity, sphingosine-1-phosphate receptor activity
A6IMB9	Taste receptor type 2	*Tas2r116*		bitter taste receptor activity, G protein-coupled receptor activity
P84889	Vang-like protein 2 (Van Gogh-like protein 2)	*Vangl2*	anterior/posterior pattern specification, apical protein localization, regulation of actin cytoskeleton organization, Wnt signaling pathway, wound healing	
A6KLQ5	Butyrophilin, subfamily 1, member A1	*Btn1a1*	regulation of cytokine production, T cell receptor signaling pathway	signaling receptor binding
D3ZYF5	Tripartite motif-containing 58	*Trim58*	innate immune response, ubiquitin-dependent protein catabolic process	ubiquitin protein ligase activity, zinc ion binding
M0R991	Natural killer cell triggering receptor	*Nktr*	protein folding	cyclosporin A binding, peptidyl-prolyl cis-trans isomerase activity
Q62751	Iron-responsive element-binding protein 2 (IRE-BP 2) (Iron regulatory protein 2) (IRP2)	*Ireb2*, *Irp2*	cellular response to iron(III) ion, cellular response to manganese ion, cellular response to mercaptoethanol, intracellular iron ion homeostasis, multicellular organismal-level iron ion homeostasis, response to copper ion, response to iron(II) ion, response to iron(III) ion, response to zinc ion	4 iron, 4 sulfur cluster binding, aconitate hydratase activity, iron-responsive element binding, metal ion binding, mRNA regulatory element binding translation repressor activity regulatory region, RNA binding, RNA binding
P97612	Fatty-acid amide hydrolase 1	*Faah*, *Faah1*	fatty acid catabolic process, fatty acid metabolic process, modulating synaptic transmission, response to ethanol, response to immobilization stress	amidase activity, fatty acid amide hydrolase activity, hydrolase activity, identical protein binding, lipid binding, phospholipid binding
D4ADD4	ADAMTS-like 3	*Adamtsl3*	extracellular matrix organization	

**Table 2:** Primer sequences of macrophage gene expression.

Target Genes	Accession Number	Primer Sequences (5′ → 3′)
*TNF-α*	NC_086038.1	F: TCTCATTCCTGCTCGTGGCG R: GGTGAGGAGCACGTAGTCG
*IL-6*	NM_012589.2	F: TTGACAGCCACTGCCTTCCC R: CGGAACTCCAGAAGACCAGAGC
*IL-10*	NM_012854.2	F: CCACATGCTCCGAGAGCTGA R: TCTTCACCTGCTCCACTGCC
*IL-12*	NM_053390.1	F: AGGTGCGTTCCTCGTAGAGA R: TTAGCCTCCACCAAGGTCAC
*IFN-γ*	NM_138880.3	F: CTGAGAGATGTTTCCCCACCG R: TCATCTGAAGTCAGCGCTCC
*β -actin*	NM_031144.3	F: CAACCTTCTTGCAGCTCCTC R: AGGGTCAGGATGCCTCTCTT

**Table 3:** PCR Conditions for macrophage gene expression.

Cycles	Temperature (°C)	Time	Notes
1	95	2 min	Polymerase activation
45	95	5 s	Denaturation
	60	10 s	Annealing
	72	20 s	Extension

## Data Availability

The MS/MS raw data and analysis are available in the ProteomeXchange Consortium via the jPOST partner repository: JPST004666 (https://repository.jpostdb.org/preview/15810794536a1f4c9577c93, access key 8086, accessed on 30 May 2026.) and PXD079178.

## Supplementary Materials

The following supporting information can be downloaded at: https://www.mdpi.com/article/10.3390/ijms27167223/s1.
