# Supplementary material for: Ag85A-PEGylated Propolis Nanoparticles Exhibit Intracellular Antimycobacterial and Host-Protective Activities Against Mycobacterium tuberculosis
Source: Int J Mol Sci. 2026 Aug 13;27(16):7223. doi: 10.3390/ijms27167223 (PMC13513740; doi:10.3390/ijms27167223)
Supplement: Supplementary file 1 [file ijms-27-07223-s001.zip › ijms-4466243-supplementary.pdf]

Supplementary data

Table S1. Physicochemical characteristics of Ag85A-PEGNio/EEP after storage in cell culture medium (15% FBS-HAMs F12K) for 2 days.

Time	Particle size (nm)	Polydispersity Index (PDI)	Zeta potential (ZP)	Encapsulation efficiency (%EE)
Day 0	220.1 ± 3.656	0.185 ± 0.019	−32.6 ± 1.80	90.38
Day 1	317.2 ± 17.32	0.450 ± 0.048	−25.5 ± 2.26	62.45
Day 2	561.2 ± 71.40	0.616 ± 0.102	−29.0 ± 1.71	55.64

Table S2. Functional enrichments in STRING protein-protein interaction (PPI) network of significant proteins identified in Mtb-infected macrophages following Ag85A-PEGNio/EEP treatment.

**Biological Process (Gene Oncology)**					
GO-term	Description	Count in network	Strength	Signal	False discovery rate
GO:0006950	Response to stress	33 of 3422	0.67	0.99	3.15e-13
GO:0009617	Response to bacterium	20 of 868	1.05	1.8	5.19e-13
GO:0051716	Cellular response to stimulus	41 of 6994	0.45	0.65	1.39e-12
GO:0071310	Cellular response to organic substance	27 of 2268	0.76	1.14	1.49e-12
GO:0006954	Inflammatory response	16 of 495	1.19	2.11	2.60e-12
GO:0002376	Immune system process	25 of 2076	0.77	1.12	1.45e-11
GO:0009987	Cellular process	46 of 15210	0.16	0.33	2.42e-06
GO:0019934	cGMP-mediated signaling	2 of 28	1.54	0.51	0.0328
**Protein Domains (SMART)**					
SM00250	Plectin repeat	2 of 6	2.21	0.48	0.0470
